# Pneumonia by *Listeria monocytogenes*: A Common Infection by an Uncommon Pathogen

**DOI:** 10.1155/2015/627073

**Published:** 2015-01-31

**Authors:** Theocharis Koufakis, Marianneta Chatzopoulou, Anastasios Margaritis, Maria Tsiakalou, Ioannis Gabranis

**Affiliations:** ^1^Department of Internal Medicine, General Hospital of Larissa, 1 Tsakalof Street, 41221 Larissa, Greece; ^2^Department of Microbiology, General Hospital of Larissa, 41221 Larissa, Greece

## Abstract

Infections by *Listeria monocytogenes* typically occur in infants, the elderly, pregnant women, and immunosuppressed subjects. Pulmonary infections in adults are extremely uncommon and only few reports can be found in the literature. We here report a case of *Listeria* pneumonia in an 85-year-old female patient and we discuss our diagnostic and therapeutic approach. Despite being rare and in most cases difficult to be identified, *Listeria* pneumonia should always be considered in immunosuppressed patients, presenting with fever and symptoms from the lower respiratory system.

## 1. Introduction

*Listeria monocytogenes* (named after the English pioneer of antiseptic surgery Joseph Lister), is a gram-positive, motile, rod-shaped bacterium. Most infections in humans are considered to be foodborne, partly due to the microorganism's property to proliferate at cooling temperatures, with cheese and unpasteurized milk being the most common sources [1]. The major form of person to person transmission is from mother to child transplacentally or at birth. It is generally considered as a rare pathogen, given that the reported incidence of* Listeria* infections in Europe is estimated to be between 0.3 (in Greece) and 7.5 (in Sweden) cases per year [2]. Listeriosis occurs primarily as bacteremia and/or meningitis, while pulmonary infections in adults are extremely uncommon [3] and only few reports can be found in the literature.

## 2. Case Presentation

**Figure 1:**
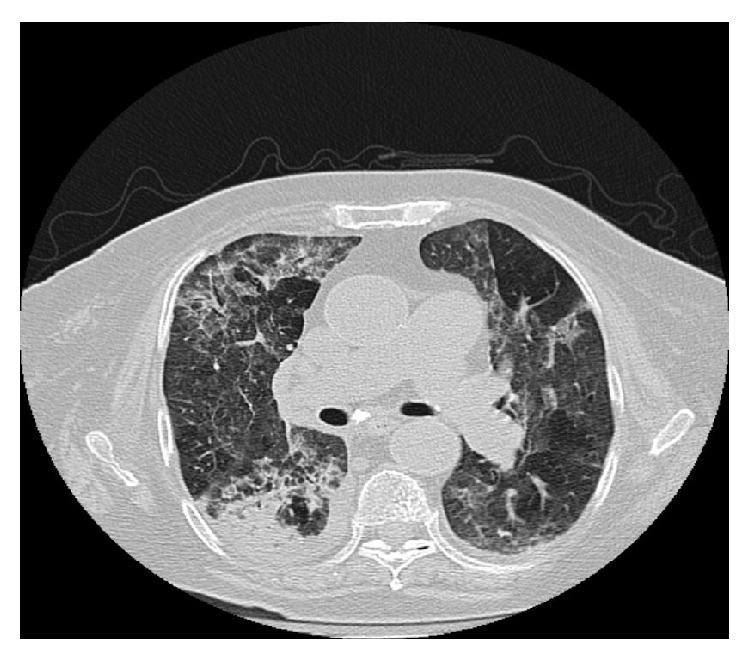
Patient's high resolution chest computed tomography demonstrating distortion of the pulmonary architecture with thickening of pulmonary interstitium and areas of ground-glass morphology, enlargement of pulmonary arteries, and right lower lobe opacities.

**Figure 2:**
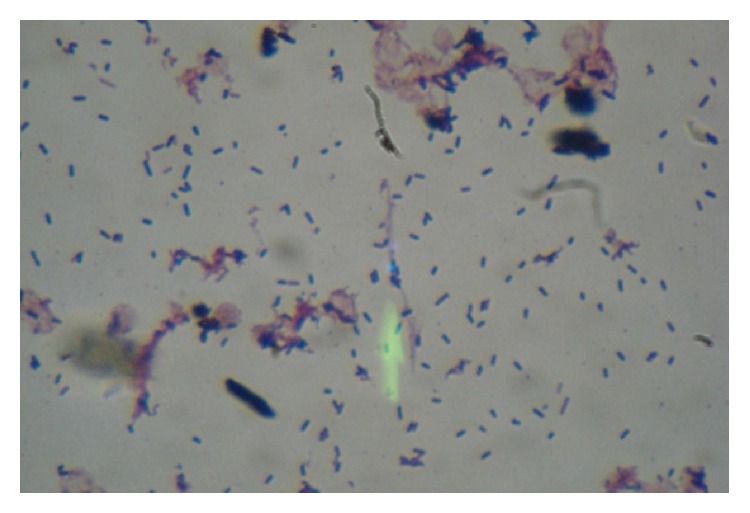
Gram stain from the positive blood culture. Numerous gram-positive rods in palisades and V-shapes.

An 85-year-old female patient presented to the emergency department with fever, cough, and dyspnea. Her symptoms began three days prior to presentation. She had a history of splenectomy after a car crash as well as of rheumatoid arthritis. She was on methotrexate (10* *mg once a week) and prednisolone (12.5 mg once a day). The patient's main clinical and laboratory findings on admission were as follows: fever (38°C), sinus tachycardia (120 beats per minute), tachypnea (30 breaths per minute), diffuse crackles heard on lung auscultation, hypoxygonemia (PO_2_ 56 mm Hg), pancytopenia (hemoglobin 9.0 g/dL, white cell count 3.0 × 10^9^/L, platelet count 120 × 10^9^/L), elevated inflammation markers (C-reactive protein 17 mg/dL, and erythrocyte sedimentation rate 90 mm/h), and abnormal glomerular filtration rate (70 mL/min/1.73 m^2^). Her chest X-ray demonstrated diffuse bilateral opacities, mainly at the right side. Further evaluation with high resolution chest computed tomography revealed distortion of the pulmonary architecture with thickening of pulmonary interstitium and areas of ground-glass morphology, as well as right lower lobe opacities (Figure 1). The overall imaging findings gave us the impression of a severe infection occurring upon a chronic respiratory disease (probably pulmonary fibrosis). Urine, blood, and sputum samples were collected and sent for culture and empiric therapy with moxifloxacin was started, as well as supportive care with fluids and oxygen. Methotrexate was withdrawn but not prednisolone, due to the possibility of adrenal insufficiency. The patient's blood culture turned positive after two days of incubation. Gram stain of the blood culture revealed numerous gram-positive rods in palisades and V-shapes resembling diphtheroids (Figure 2). Subsequent subculture revealed catalase-positive, esculin-positive, and gram-positive rods growing on 5% sheep blood agar and chocolate agar and showing weak beta-hemolysis. The microorganism was identified as* Listeria monocytogenes* with Vitek 2 automated system. The microorganism was tested susceptible to benzylpenicillin, ampicillin, meropenem, and erythromycin and resistant to cotrimoxazole according to the breakpoints set by EUCAST. No pathogenic organism was isolated from the respiratory culture. The gram stain of the sputum revealed superficial contamination and due to the presence of gram-positive rods along with other members of the normal oropharyngeal flora the specimen was regarded as inconclusive about the potential pathogen. Thereafter, the antibiotic therapy was changed to ampicillin (12 grams per day), according to antibiogram results (minimum inhibitory concentration < 1). Patient's exposure to any source of the microorganism could not be documented. Fever's remission was observed 24 hours after ampicillin was started and, within five days, the patient's clinical, laboratory, and imaging findings were slightly improved. Seven days after admission she developed massive hematochezia and hemodynamic instability. Urgent gastroscopy was negative for active source of bleeding, while colonoscopy revealed multiple sigmoid colon diverticula. Surgical intervention was considered to be extremely risky, because of existing comorbidities and patient's general condition. She died 48 hours later due to hypovolemic shock, despite having been given intensive supportive care.

## 3. Discussion

Infections by* Listeria monocytogenes* typically occur in infants, the elderly, pregnant women, and immunosuppressed subjects and are characterized by an overall mortality rate, believed to be between 15% and 30% [4]. With regards to neonatal pneumonia*, Listeria* is included among the pathogens most frequently involved [5].

Diagnosis is based mainly on the isolation of the pathogen from infected tissues and fluids, while imaging, histopathological, and other laboratory methods are of limited use, due to the fact that listeriosis does not present with specific signs and symptoms. In our case, blood cultures proved to be positive for* Listeria* but not the sputum ones. Generally, the clinical utility of the sputum culture has been debated and the reported sensitivity varies significantly among different studies. Sputum has a low diagnostic yield and does not contribute significantly to patient management [6]. Our patient presented with high fever and respiratory symptoms. The radiological findings revealed diffuse bilateral findings especially in the lower lobes where blood preferentially flows. This is more consistent with hematologic spread of the pathogen to the lungs rather than inhalation which would probably lead to a focal inflammation. The positive blood cultures confirmed our hypothesis and revealed an unusual pathogen.

In general,* Listeria* species are susceptible to antibiotics of choice; still, resistant strains isolated from human infections have been described [7]. In this particular case, the microorganism was found to be cotrimoxazole resistant, which is generally uncommon [7]. This is of great clinical significance, given that cotrimoxazole is often listed as the empiric antibiotic agent of choice for the treatment of* Listeria* infections in penicillin allergic patients [8].

When gram-positive rods are recovered from clinical specimens, they are usually regarded as contaminants. Nevertheless, upon their recovery from immunosuppressed patients physicians should suspect a possible* Listeria* infection. This is clinically important due to the species' intrinsic resistance to wide-spectrum cephalosporins [9], which are widely used as first-line empiric treatment in central nervous system, bloodstream, and pulmonary infections. Ampicillin in combination with an aminoglycoside is generally considered as the optimal therapy. In our case, treatment only with ampicillin based on the sensitivity report was preferred, due to aminoglycosides' potential nephrotoxicity.

*Listeria monocytogenes* is a facultative intracellular pathogen. Therefore, impaired cell-mediated immunity is believed to be a predisposing condition for infection. The murine model of listeriosis has significantly contributed to the understanding of the cellular immune response in* Listeria* infection in general, as well as in* Listeria* pneumonia more specifically [10]. The particular patient belonged to a high-risk group due to her age and due to immunosuppression with corticosteroids and methotrexate. She had, also, been subjected to splenectomy. The patient's death occurred due to a condition apparently irrelevant to the* Listeria* infection. However, the possibility that the overall inflammation stress contributed to the development of intestinal bleeding cannot be excluded.

In conclusion, despite being rare and in most cases difficult to be identified,* Listeria* pneumonia should always be considered in immunosuppressed patients, presenting with fever and symptoms from the lower respiratory system.
